# Genome-Wide Transcription and Functional Analyses Reveal Heterogeneous Molecular Mechanisms Driving Pyrethroids Resistance in the Major Malaria Vector *Anopheles funestus* Across Africa

**DOI:** 10.1534/g3.117.040147

**Published:** 2017-04-19

**Authors:** Jacob M. Riveron, Sulaiman S. Ibrahim, Charles Mulamba, Rousseau Djouaka, Helen Irving, Murielle J. Wondji, Intan H. Ishak, Charles S. Wondji

**Affiliations:** * Liverpool School of Tropical Medicine, L3 5QA, United Kingdom; † Organisation de Coordination pour la lutte contre les Endémies en Afrique Centrale (OCEAC), P.O. Box 288 Yaoundé, Cameroon; ‡ Department of Biochemistry, Bayero University, PMB 3011 Kano, Nigeria; § Uganda Virus Research Institute, P.O. Box 49 Entebbe, Uganda; ** International Institute of Tropical Agriculture, Cotonou 08 BP 0932, Benin; †† School of Biological Sciences, Universiti Sains Malaysia, Gelugor, 11800 Penang, Malaysia

**Keywords:** Malaria, *Anopheles funestus*, pyrethroids, insecticide resistance, cytochrome P450

## Abstract

Pyrethroid resistance in malaria vector, *An. funestus* is increasingly reported across Africa, threatening the sustainability of pyrethroid-based control interventions, including long lasting insecticidal nets (LLINs). Managing this problem requires understanding of the molecular basis of the resistance from different regions of the continent, to establish whether it is being driven by a single or independent selective events. Here, using a genome-wide transcription profiling of pyrethroid resistant populations from southern (Malawi), East (Uganda), and West Africa (Benin), we investigated the molecular basis of resistance, revealing strong differences between the different African regions. The duplicated cytochrome P450 genes (*CYP6P9a* and *CYP6P9b*) which were highly overexpressed in southern Africa are not the most upregulated in other regions, where other genes are more overexpressed, including *GSTe2* in West (Benin) and *CYP9K1* in East (Uganda). The lack of directional selection on both *CYP6P9a* and *CYP6P9b* in Uganda in contrast to southern Africa further supports the limited role of these genes outside southern Africa. However, other genes such as the P450 *CYP9J11* are commonly overexpressed in all countries across Africa. Here, *CYP9J11* is functionally characterized and shown to confer resistance to pyrethroids and moderate cross-resistance to carbamates (bendiocarb). The consistent overexpression of *GSTe2* in Benin is coupled with a role of allelic variation at this gene as GAL4-UAS transgenic expression in *Drosophila* flies showed that the resistant 119F allele is highly efficient in conferring both DDT and permethrin resistance than the L119. The heterogeneity in the molecular basis of resistance and cross-resistance to insecticides in *An. funestus* populations throughout sub-Saharan African should be taken into account in designing resistance management strategies.

Malaria remains one of the main causes of morbidity and mortality in Sub-Saharan Africa, predominantly in children under 5 yr and pregnant mothers (WHO 2015). *Anopheles funestus* s.s. is one of the major malaria vectors in Sub-Saharan Africa and is widely distributed across the continent (Gillies and De Meillon 1968). The important role of *An. funestus* in malaria transmission is supported by recent reports indicating high *Plasmodium falciparum* parasite infection rates in this vector in many Sub-Saharan countries (Coetzee and Koekemoer 2013; Dia *et al.* 2013). Malaria vector control relies heavily on the use of a single insecticide class—the pyrethroids. Pyrethroids are safe and fast acting (Zaim *et al.* 2000), and are the only class of insecticides approved for use on insecticide treated materials such as Long Lasting Insecticide Nets (LLINs) (http://www.who.int/whopes/en/). As in other malaria vectors, pyrethroid resistance in *An. funestus* has increasingly been reported in Sub-Saharan Africa from different regions, including southern [South Africa (Hargreaves *et al.* 2000; Brooke *et al.* 2001), Mozambique (Casimiro *et al.* 2006; Cuamba *et al.* 2010), Malawi (Hunt *et al.* 2010; Wondji *et al.* 2012)], East [Uganda and Kenya (Morgan *et al.* 2010; Mulamba *et al.* 2014) and Tanzania (Lwetoijera *et al.* 2014)], Central [Cameroon (Wondji *et al.* 2011; Menze *et al.* 2016)], or West Africa [Benin (Djouaka *et al.* 2011, R. Djouaka *et al.* 2016), Ghana (Okoye *et al.* 2008; Riveron *et al.* 2016), Senegal (Samb *et al.* 2016) and Nigeria (Ibrahim *et al.* 2014; R. J. Djouaka *et al.* 2016)]. These increasing reports of pyrethroid resistance in malaria vectors such as *An. funestus* is of great concern as it poses serious threats to the effectiveness of the malaria vector control tools across the continent (WHO 2012). Thus, the urgent calls to develop and implement suitable resistance management strategies against malaria vectors, to ensure sustainable effectiveness of malaria vector control interventions. Understanding the molecular basis of insecticide resistance in malaria vectors is critical for designing and implementing these resistance management strategies.

Cases of pyrethroids resistance reported so far in *An. funestus* populations are mainly caused by metabolic resistance mechanisms, with no evidence of target-site resistance through knockdown resistance (*kdr*) (Amenya *et al.* 2008; Okoye *et al.* 2008; Wondji *et al.* 2012; Riveron *et al.* 2013). Cytochrome P450s are known to be the primary enzyme family conferring resistance to pyrethroids. Molecular studies conducted in southern Africa, notably in Malawi and Mozambique, have revealed that the duplicated P450 genes, *CYP6P9a*, and *CYP6P9b* are the main genes driving pyrethroid resistance in this species in this region (Amenya *et al.* 2008; Wondji *et al.* 2009; Riveron *et al.* 2013). However, studies performed in Zambia suggested a diminishing role of these two duplicated P450s northward (Riveron *et al.* 2014a; Thomsen *et al*. 2014). Furthermore, a recent study has revealed a similar minor role of *CYP6P9a* and *CYP6P9b* across a south-north transect in Malawi, with low expression of these two genes in the north in contrast to high levels in the south, coupled with a nearly fixed resistant haplotype (Barnes *et al.* 2016). This variation of expression profiles in southern Africa suggests that there could also be significant differences in the underlying genetic drivers of pyrethroid resistance across African populations of *An. funestus*. However, the molecular basis of pyrethroid resistance in *An. funestus* in other African regions, such as in East or West Africa, remains poorly characterized despite the high level of pyrethroid resistance also reported in these regions (Okoye *et al.* 2008; Morgan *et al.* 2010; Djouaka *et al.* 2011; Mulamba *et al.* 2014).

Here, using a microarray genome-wide transcription analysis, we characterized the molecular basis of pyrethroid resistance in this major vector in West and East Africa and through a comparative analysis with southern African populations, we revealed sharp difference in the key genes driving resistance in each region. The P450 *CYP9J11* commonly overexpressed in all countries was functionally characterized, and shown to confer resistance to pyrethroids and moderate cross-resistance to carbamates. In addition, allelic variation in the glutathione S-transferase gene, *GSTe2*, through the L119F mutation (Riveron *et al.* 2014b) was established to be playing a major role in both DDT and pyrethroid resistance in Benin.

## Materials and Methods

### Study sites and samples

Blood-fed (F_0_) females resting indoors were collected between 06.00 am and 12.00 pm in Tororo, Eastern Uganda (0.69° N, 34.18° E), in July 2012. Benin samples were collected in Pahou (6° 23′ N, 2° 13′ E) in southern Benin, West Africa in April 2011. The Malawian samples were collected in the Chikwawa District (0° 45′ N, 34° 5′E) in southern Malawi between July 2009 and April 2010. The F_0_ collection method and F_1_ rearing were conducted as described previously (Djouaka *et al.* 2011; Riveron *et al.* 2013; Mulamba *et al.* 2014). All F_0_ adults used for individual oviposition of the above F_1_ eggs were morphologically identified as belonging to the *An. funestus* group according to the key of Gillies and Coetzee (1987). A PCR assay was performed using the protocol of (Koekemoer *et al.* 2002) to confirm that collected F_0_ adults were *An. funestus* s.s. The study samples were 2- to 5-d-old F_1_ adult permethrin-resistant *An. funestus* s.s. mosquitoes.

### Resistance profile of different populations

The resistance patterns of the three populations to various insecticides was determined as described previously (Djouaka *et al.* 2011; Riveron *et al.* 2013; Mulamba *et al.* 2014) following the World Health Organization (WHO) protocol (WHO 1998). The Pahou populations of *An. funestus* from Benin is highly resistant to DDT (0% mortality after 1 hr exposure), resistant to both Type I (permethrin; 66% mortality) and II (deltamethrin; 88% mortality) pyrethroid, resistant to carbamates (bendiocarb; 64% mortality), but fully susceptible to malathion (Djouaka *et al.* 2011). The Uganda population from Tororo is resistant to pyrethroids [permethrin (33% mortality), deltamethrin (20% mortality)] and DDT (61% mortality), but susceptible to other insecticide classes (Mulamba *et al.* 2014). The Malawi population from Chikwawa in 2010 was resistant to pyrethroid [permethrin (47.2% mortality), deltamethrin (42.3% mortality)] and carbamates (bendiocarb; 60% mortality), moderately resistant to DDT (87.8% mortality), and fully susceptible to organophosphates (Wondji *et al.* 2012; Riveron *et al.* 2013).

### Detection of pyrethroid resistance genes using microarrays

The 8 × 60k Agilent microarray chip custom designed for *An. funestus* used for this study was previously described (Riveron *et al.* 2014a). Briefly, each chip contains 60mer probes designed from *An. funestus* published ESTs from transcriptome sequencing by 454 (8540) (Gregory *et al.* 2011), Illumina (15,527) (Crawford *et al.* 2010), or *An. funestus* cDNAs from GenBank (2850) (two probes for each EST). It also includes a set of P450 genes from the *rp1* and *rp2* QTL genomic regions (Wondji *et al.* 2009; Irving *et al.* 2012) (three probes for each gene), the complete set of *Anopheles gambiae* transcripts (13,000) (one probe each), and all of the *An. gambiae* detoxification genes (David *et al.* 2005) (three probes for each gene). In Benin, we also used the other 4 × 44k *An. funestus* chip (A-MEXP-2245), previously described (Riveron *et al.* 2013) in a triangular experimental design comparing resistant (R), control (C), and susceptible (S) samples.

The Picopure RNA Isolation Kit (Arcturus) was used to extract total RNA from three biological replicates, each made of batches of 10 2- to 5-d-old F_1_ *An. funestus* from each field sample that had survived exposure to 0.75% permethrin for 1 hr (R). The same was done also for the fully susceptible laboratory strain FANG (S). Mosquitoes from Benin not exposed to insecticide (C) were also extracted. The RNA extraction was performed as previously described (Riveron *et al.* 2014a). Complementary RNA (cRNA) was amplified from each sample using the Agilent Quick Amp Labeling Kit (two-color) following the manufacturer’s protocol. The cRNA samples from the susceptible strain FANG (S) were labeled with the cy3 dye, and cRNAs from the resistant samples (R) were labeled with cy5 dye. The cRNA quantity and quality were assessed before labeling using the NanoDrop and Bioanalyzer. Labeled cRNAs were hybridized to the arrays for 17 hr at 65° according to the manufacturer’s protocol. Five hybridizations were performed for each sample by swapping the biological replicates. Agilent GeneSpring GX 13.0 software was used to analyze the microarray data. The differentially expressed genes were identified using a threshold of twofold-change (FC) and a statistical significance of *P* < 0.01 with Benjamini-Hochberg correction for multiple testing. The BLAST2GO program was used to predict the functions of all the transcripts used to design the microarray chip (Conesa *et al.* 2005; Gotz *et al.* 2008). Gene Ontology (GO) enrichment analyses were preformed using BLAST2GO to detect the major GO terms over-represented among the sets of probes upregulated in various hybridizations and countries in comparison to the reference set made of the entire transcript set on the microarray chip. The Fisher’s test was used to assess the statistical significance of these tests.

### Quantitative RT-PCR validation of the candidate resistance genes

Quantitative reverse transcription PCR (qRT-PCR) assays were performed to validate microarray results for the key candidate genes; 1 µg of RNA from each of the three biological replicates—the Resistant (R), Control (C), and FANG (S)—was used as a template for complementary (cDNA) synthesis using the superscript III (Invitrogen) following the manufacturer’s guide. The qRT-PCR was carried out as previously described (Kwiatkowska *et al.* 2013; Riveron *et al.* 2013) with the relative expression level and FC of each target gene in R and C relative to S calculated according to the 2^−ΔΔCT^ method (Schmittgen and Livak 2008) after normalization with the housekeeping genes ribosomal protein S7 (*RSP7*; AFUN007153) and actin5C (AFUN006819). The primers are listed in Supplemental Material, Table S1 in File S1.

### Heterologous expression of candidate genes in Escherichia coli

#### Cloning of CYP9J1 for expression in E. coli:

The full-length *CYP9J11* was amplified from cDNAs (used for qRT-PCR) and cloned into the pJET1.2/blunt cloning vector (Thermo Scientific). The primers used are listed in Table S1 in File S1 as CYP9J11Full F and R. After sequence analysis, one clone predominant in the three countries was selected for functional characterization. This *CYP9J11* allele was fused to a bacterial ompA+2 leader sequence, and expressed in *E. coli* JM109 cells using the pCW-ori+ vector as previously described (Pritchard *et al.* 1998; McLaughlin *et al.* 2008; Stevenson *et al.* 2011). Briefly, a DNA fragment containing the coding sequence for the ompA+2 signal peptide with a downstream alanine-proline linker, and the first ∼20 nt of *CYP9J11* was first amplified using 50 ng of *E. coli* JM109 DNA as the template (Primer sets in Table S1 in File S1). Next, this *CYP9J11* clone and the ompA+2 PCR fragment were used as templates in a fusion PCR under the same conditions described previously (Riveron *et al.* 2014a). The full-length sequence of *CYP9J11* incorporating the ompA+2 leader was ligated into a modified pCW-ori+ vector plasmid, pB13 (Pritchard *et al.* 1998), via *EcoR*I and *XBa*I sites to produce pB13::ompA+2-CYP9J11. This construct was also sequenced to confirm the absence of PCR errors.

#### Membrane preparation:

Membranes containing CYP9J11 were obtained by cotransforming the *E. coli* cells JM109 with pB13::ompA+2-CYP9J11 with a plasmid containing the *An. gambiae* cytochrome P450 reductase, pACYC-AgCPR (McLaughlin *et al.* 2008). The expression of CYP9J11, membrane isolation, and determination of P450 content were carried out as previously described (McLaughlin *et al.* 2008; Stevenson *et al.* 2011). The membranes were stored in aliquots at −80°, and assayed for total protein concentration using NanoDrop spectrophotometer, P450 concentration (Omura and Sato 1964) and CPR activity by monitoring *cytochrome c* reduction (Strobel and Dignam 1978). The histidine-tagged *An. gambiae* cytochrome *b*_5_ was generated as previously described by Stevenson *et al.* (2011) and used for the metabolism assays.

#### Metabolism assays:

*In vitro* metabolism reactions between pyrethroids (deltamethrin and permethrin) and carbamates (bendiorcab and propuxur) and membranes expressing CYP9J11 were performed as previously described (Stevenson *et al.* 2011; Ibrahim *et al.* 2016a) in the presence of CPR with cytochrome *b*_5_. The reactions consisted of the following: 45 pmol of P450, 0.2 M Tris HCl pH 7.4, 0.25 mM MgCl_2_, 1 mM glucose-6-phosphate, 0.1 mM NADP+ (Melford), 1 unit/ml glucose-6-phosphate dehydrogenase (G6PDH), 0.8 µM cytochrome *b*_5_, and 0.2 mM of test insecticide in a final volume of 200 ml.

#### HPLC analysis:

Detection of the reaction outcome followed standard protocol (Stevenson *et al.* 2012), with the reactions stopped by addition of 0.1 ml ice-cold methanol and incubation for 5 min with shaking to dissolve all available pyrethroids. After a centrifugation of the samples, 150 µl of the supernatant was transferred into HPLC vials; 100 µl sample was loaded into an isocratic mobile phase of 90% methanol and 10% water with a flow-rate of 1 ml/min, and substrate peaks were separated on a 250 mm C18 column (Acclaim 120, Dionex) at 23°. The quantity of pyrethroid remaining in the samples was determined by reverse-phase HPLC with a monitoring absorbance wavelength of 226 nm (Agilent 1260 Infinity). Percentage depletion was calculated by comparing the area of the chromatogram from incubation with NADPH regeneration system to the tubes in which NADP^+^ was not added (NADP^−^). HPLC conditions for analysis of the nonpyrethroid insecticides was as described in a previous study (Ibrahim *et al.* 2016b).

#### Turnover and kinetic assays:

To determine the turnover of CYP9J11 with pyrethroids and bendiocarb, experiments with deltamethrin, permethrin, and bendiocarb were performed in with incubation time varied from 0 to 30 min. For kinetic constants, incubation was carried out with 20 µM each of deltamethrin, permethrin, and bendiocarb for 30 min. The turnover and steady-state kinetic parameters (K_m_ and V_max_) were calculated as previously described (Ibrahim *et al.* 2015) using the enzyme kinetic module of GraphPad Prism 6.03 (GraphPad Software Inc., La Jolla, CA). Catalytic constants and efficiencies were determined from the steady-state parameters.

### Transgenic expression of candidate genes in Drosophila strains and tests with insecticides

To functionally validate the role of *An. funestus CYP9J11* (an ortholog of *CYP9J5* in *An. gambiae*) in conferring pyrethroid resistance (*CYP9J11* is consistently overexpressed in all three countries), transgenic *Drosophila melanogaster* flies expressing this gene were generated using the GAL4/UAS system. This is to establish whether *CYP9J11* overexpression alone could confer resistance to pyrethroids. The construction of the transgenic strain followed the protocol we used successfully for the P450s *CYP6P9a* and *CYP6P9b* (Riveron *et al.* 2013), and *CYP6M7* (Riveron *et al.* 2014a).

Briefly, the same predominant clone used for transgenic expression was selected to construct transgenic flies, and cloned into the pUASattB vector using primers containing restriction sites for *bglII* and *Xba*I (see Table S1 in File S1). The PhiC31 system was used to generate the transgenic line UAS-CYP9J11 by Genetic Services (Cambridge, MA). Ubiquitous expression of the transgene *CYP9J11* in adult F_1_ progeny (experimental group) was obtained after crossing virgin females from the driver strain Act5C-GAL4 [“y[1] w[*]; P(Act5C-GAL4-w)E1/CyO,”“1;2”] (Bloomington Stock Center, Bloomington, IN) with homozygote UAS-CYP9J11 males. Similarly, adult F_1_ control progeny (control group) with the same genetic background as the experimental group but without expression of *CYP9J11* were obtained by crossing virgin females from the driver strain Act5C-GAL4 and UAS recipient line males (which do not carry the pUASattb-CYP9J11 insertion).

Insecticide contact bioassays for both experimental and control F_1_ *Drosophila melanogaster* females were performed as previously described (Riveron *et al.* 2014a) using posteclosion females that were 2–5 d old for contact assay with the pyrethroids deltamethrin (0.15%) and permethrin (2%)-impregnated filter papers prepared in acetone and Dow Corning 556 Silicone Fluid (BHD/Merck, Germany). A total of 20–25 flies was placed in individual vial containing respective insecticide papers, and the mortality plus knockdown was scored after 1, 2, 3, 6, 12 24, 36, and 48 hr of exposure to the insecticide. For all assays, at least six replicates were performed. Student’s *t*-test was used to compare the mortality plus knockdown of the experimental group against the control group.

#### Investigating the role of allelic variation at GSTe2 in the permethrin resistance:

Due to the overexpression of *GSTe2* in mosquitoes resistant to permethrin in Benin, we used the transgenic expression in *Drosophila* to assess whether the allelic variation observed at this gene with the L119F mutation was playing a role in the observed resistance. A transgenic line was generated using the susceptible L119-GSTe2 allele following the same protocol described previously for the resistant allele 119F-GSTe2 as well as for the bioassays with permethrin and deltamethrin, but also DDT (Riveron *et al.* 2014b). Student’s *t*-test was used to compare the mortality plus knockdown of the L119-GSTe2 group against the control group and 119F-GSTe2 group.

### Genetic diversity of candidate resistance genes between different An. funestus populations from different regions of Africa

#### Genetic variability of CYP9J11:

The full-length coding region of *CYP9J11* was amplified from cDNA of permethrin-resistant samples from Malawi, Uganda and Zambia to assess the polymorphism of this gene. The Zambia mosquitoes were collected in Katete district (14° 11′ 0′′ S, 31° 52′ 0′′ E) in 2010 as previously described (Riveron *et al.* 2014a). The amplification was performed using the same cDNA synthesized for qRT-PCR with the Phusion polymerase (Thermo Scientific), which was cloned and sequenced as described above.

#### Comparative genetic diversity of CYP6P9a and CYP6P9b between East and southern Africa:

To assess whether previously detected directional selection associated with high overexpression of *CYP6P9a* and *CYP6P9b* genes in southern Africa was also present in East Africa, mosquitoes from Tororo in Uganda were compared to those from Chikwawa in Malawi (Riveron *et al.* 2013). Genomic fragment of both genes spanning the full-length coding region and a portion of the 5′UTR region were amplified and sequenced directly in 10 susceptible (dead after 1 hr exposure) from Tororo and 10 resistant mosquitoes (alive after 1 hr exposure) to 0.75% permethrin. The primers used are listed in Table S1 in File S1. Polymorphic positions were detected through manual analysis of sequence traces using BioEdit, and as sequence differences in multiple alignments using ClustalW (Thompson *et al.* 1994). DnaSP 5.1 (Rozas *et al.* 2003) was used to define the haplotype phase (through the Phase program), and to assess genetic parameters of each gene such as nucleotide diversity (π) and haplotype diversity. A maximum likelihood phylogenetic tree of the haplotypes for each gene was constructed using MEGA 5.2 (Tamura *et al.* 2007) to assess the potential correlation between haplotypes and resistance phenotypes.

### Data availability

The microarray data from this study were submitted to Array Express, accession numbers E-MTAB-5375, E-MTAB-5376, and E-MTAB-5424. The DNA sequences reported in this paper have been deposited in the GenBank database (accession numbers: KJ150626–KJ150674).

## Results

### Transcription profiling of the pyrethroid resistant population of Uganda

**Figure 1:**
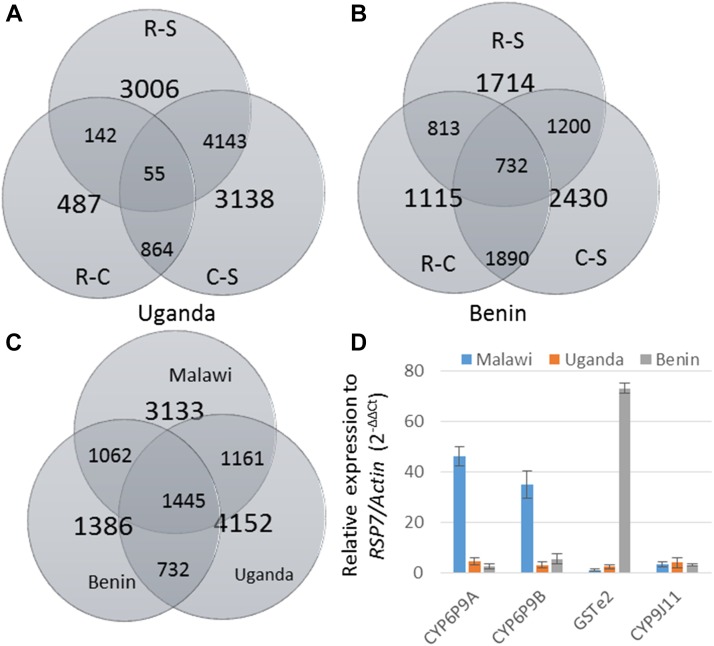
Transcription profile of pyrethroid resistance across Benin, Uganda, and Malawi: (A, B) Venn diagram summarizing the number of probes differentially expressed in each and between comparisons in (A) Uganda and (B) in Benin at fold-change (FC) >2 and *P* < 0.05 in R-S, C-S and R-C comparisons, as well as the commonly expressed probes. (C) Venn-diagram of the comparison between Malawi, Uganda, and Benin for the R-S comparison only. (D) qRT-PCR expression of key resistance genes in the three countries when comparing the permethrin-resistant mosquitoes to FANG susceptible (R-S) mosquitoes.

To detect the set of genes associated with permethrin resistance in Uganda, the 8 × 60k microarray chip was used to compare mosquitoes alive to permethrin exposure to control (nonexposed) (R-C) and to the fully susceptible laboratory strain FANG (R-S). The control mosquitoes were also compared to the susceptible FANG strain (C-S). High numbers of probes were significantly differentially expressed (*P* < 0.05) for the R-S (7346) and C-S (7479) comparisons (Figure 1A), most likely due to extensive genetic differences between the samples. In contrast, for the R-C comparison, a lower number (827) of differentially expressed probes was observed, as described previously in other similar studies (Riveron *et al.* 2013) due to the high level of resistance in the population. Consequently, only 55 probes were commonly differentially expressed in all three comparisons.

#### R-S/C-S/R-C:

The cytochrome P450 *CYP4C27* [Afun012777 using the ID system set by Crawford *et al.* (2010)] was the only detoxification gene commonly overexpressed in R-C, R-S and C-S, with highest fold change in R-S (FC10.3) (Table 1). Other genes had no annotation or no previous association with insecticide resistance such as an *acyl-oxidase* Afun004337 (AGAP011798-RA).

**Table 1:** List of top detoxification genes significantly overexpressed in pyrethroid resistant *An. funestus* in Uganda for all comparisons

Probe	Transcript	*An. gambiae* ID	R-C	R-S	C-S	Description
CUST_12777_PI426302897	Afun012777 CYP4C27	AGAP009246-PA	3.6	10.3	6.8	Cytochrome p450
CUST_8293_PI426302897	Afun008293	AGAP008291-PA		187.8	83.0	Trypsin-related protease
CUST_7663_PI426302897	Afun007663 (CYP6M7)	AGAP008213-PA		70.1	24.0	Cytochrome p450
CUST_13921_PI426302897	Afun013921	AGAP006709-PA		64.4	49.5	Chymotrypsin 1
CUST_500_PI426302897	Afun000500	NA		27.1	23.0	Glycogenin
CUST_9227_PI426302897	Afun009227	AGAP008141-PA		22.8	21.2	Argininosuccinate lyase
CUST_7769_PI426302897	Afun007769 (CYP9K1)	AGAP000818-PA		21.5	16.1	Cytochrome p450
CUST_15331_PI426302897	Afun015331 (CYP307A1)	AGAP001039-PB		16.6	3.9	Cytochrome p450
CUST_11042_PI426302897	Afun011042	AGAP003321-PA		13.3	8.1	Glycine dehydrogenase
CUST_13870_PI426302897	Afun013870	AGAP012697-PA		11.9	14.8	Sulfotransferase
CUST_295_PI406199798	AGAP000177-RA	AGAP000177-RA		10.5	8.0	Cuticle protein 7
CUST_4223_PI426302897	Afun004223 (CYP4H17)	AGAP008358-PA		10.1	7.5	Cytochrome p450
CUST_15523_PI426302897	Afun015523	AGAP010581-PA		8.1	5.7	abc transporter
CUST_4631_PI406199798	AGAP005698-RA	AGAP005698-RA		7.6	3.5	Cuticular protein 4
CUST_1458_PI406199769	combined_c738			6.7	4.9	Short-chain dehydrogenase
CUST_13481_PI426302897	Afun013481 (GSTe1)	AGAP009195-PA		6.6	5.6	Glutathione-s-transferase
CUST_3246_PI426302897	Afun003246	AGAP006220-PA		6.5	4.5	Aldehyde oxidase
CUST_8354_PI426302897	Afun008354 (GSTD3)	AGAP004382-PA		6.5	5.1	Glutathione transferase
CUST_12343_PI426302897	Afun012343 (CYP4H17)	AGAP008358-PA		6.0	4.4	Cytochrome p450 4d1
CUST_11963_PI426302897	Afun011963	AGAP006220-PA		5.7	4.0	Aldehyde oxidase
CUST_11037_PI426302897	Afun011037	AGAP003581-PA		5.7	8.0	Alcohol dehydrogenase
CUST_376_PI406199788	gb-CYP4H25			5.4	5.3	Cytochrome p450
CUST_12197_PI426302897	Afun012197 (CYP304B1)	AGAP003066-PA		5.2	3.3	Cytochrome p450
CUST_7127_PI426302897	Afun007127 (CYP4C36)	AGAP009241-PA		5.2	2.6	Cytochrome p450
CUST_6930_PI426302897	Afun006930 (CYP6M2)	AGAP008212-PA		5.1	5.3	Cytochrome p450
CUST_7861_PI426302897	Afun007861 (CYP6Z1)	AGAP008219-PA		4.8	2.5	Cytochrome p450
CUST_10949_PI426302897	Afun010949	AGAP010887-PA		4.6	7.4	Cuticular protein rr-1 family
CUST_7696_PI406199798	AGAP008141-RA	AGAP008141-RA		4.6	2.2	Argininosuccinate lyase
CUST_3731_PI406199772	CD577517.1			4.2	4.7	Cuticle protein
CUST_7369_PI426302897	Afun007369 (CYP6P9a)	AGAP002865-PA		4.2	3.0	Cytochrome p450
CUST_13871_PI426302897	Afun013871	AGAP012697-PA		4.1	2.3	Sulfotransferase
CUST_13273_PI406199769	combined_c6791 (CYP9J11)	AGAP012296-PA		4.1	3.6	Cytochrome p450
CUST_12461_PI426302897	Afun012461	AGAP000288-PA		4.1	6.8	Alcohol dehydrogenase
CUST_7722_PI426302897	Afun007722	AGAP009850-PA		4.0	3.6	Abc transporter
CUST_10360_PI426302897	Afun010360	AGAP006222-PA		4.0	3.2	UDP glucosyl transferases
CUST_9866_PI426302897	Afun009866 (GSTe5)	AGAP009192-PA		3.9	2.7	Glutathione-s-transferase
CUST_9492_PI426302897	Afun009492	AGAP001722-PA		3.8	8.8	Carboxylesterase
CUST_7469_PI426302897	Afun007469 (CYP9J11)	AGAP012296-PA		3.8	3.0	Cytochrome p450
CUST_15244_PI426302897	Afun015244	AGAP000820-PA		3.7	5.9	Cuticular protein rr-1 family
CUST_10836_PI426302897	Afun010836	AGAP006228-PA		3.4	2.3	Esterase b1
CUST_484_PI406199788	gb-CYP9J3			3.3	2.1	Cytochrome p450
CUST_12666_PI426302897	Afun012666 (CYP315A1)	AGAP002429-PA		3.2	3.7	Cytochrome p450
CUST_405_PI406199788	gb-CYP6AD1			3.2	2.0	Cytochrome p450
CUST_9027_PI426302897	Afun009027	AGAP009463-PA		3.1	2.1	Abc transporter
CUST_9335_PI426302897	Afun009335	AGAP003343-PA		3.1	2.8	Cytochrome p450
CUST_720_PI406199788	gb-PX4B			3.1	2.8	Oxidase peroxidase
CUST_10630_PI426302897	Afun010630 (CYP6P5)	AGAP002866-PA		3.1	6.3	Cytochrome p450
CUST_45_PI426302897	Afun000045 (GSTe2)	AGAP009194-PA		2.9	2.1	Glutathione-s-transferase gst
CUST_10994_PI426302897	Afun010994 (CYP6P4)	AGAP002867-PA		2.8	3.2	Cytochrome p450
CUST_30_PI426302915	CYP6Z3			2.8	2.4	Cytochrome p450
CUST_3315_PI406199769	combined_c1675			2.7	2.6	UDP glucosyl transferases
CUST_8909_PI426302897	Afun008909 (CYP4K2)	AGAP002416-PA		2.7	3.0	Cytochrome p450
CUST_35_PI406199775	COEAE6O	AGAP002863-PA		2.6	3.1	Carboxylesterase
CUST_7499_PI426302897	Afun007499 (GSTD1-5)	AGAP004164-PA		2.5	2.1	Glutathione transferase
CUST_9584_PI426302897	Afun009584 (CYP6M4)	AGAP008214-PA		2.3	3.2	Cytochrome p450
CUST_3394_PI426302897	Afun003394 (CYP325A1)	AGAP000284-PA		2.1	2.1	Cytochrome p450

#### R-S/C-S only:

Among the most overexpressed genes commonly observed in R-S and C-S were proteases such as a trypsin-related protease (Afun008293), which was the top upregulated with FC187.8 in R-S and 83.07 in C-S. Other highly overexpressed proteases included chymotrypsin 1 (Afun013921), with FC64.4 in R-S and 49.5 in C-S. High overexpression of proteases is commonly reported in resistant mosquitoes either *Anopheles* (Kwiatkowska *et al.* 2013; Riveron *et al.* 2013), or *Aedes albopictus* (Ishak *et al.* 2016). Several detoxification genes were commonly upregulated in both comparisons, with a predominance of cytochrome P450s, notably *CYP6M7* (Afun007663), which was the most overexpressed with FC70.1 in R-S and 24 in C-S. This P450 has previously been shown to metabolize pyrethroids (Riveron *et al.* 2014a). Other highly overexpressed P450 genes included *CYP9K1* (Afun007769) with a higher fold change (FC21.5) in R-S than previously observed in southern Africa, suggesting a higher role played by this gene in Uganda. The *CYP307A1* (Afun015331) exhibited a high FC in R-S (FC16.6). Other cytochrome P450s included CYP6 subfamily genes such as *CYP6Z1*, *CYP6P5*, *CYP6P4*, *CYP6Z3*, and, notably, the *CYP6M8* (Afun006930), of which the ortholog from *An. gambiae*, *CYP6M2* is responsible for pyrethroid resistance in this species (Stevenson *et al.* 2011; Mitchell *et al.* 2012) but was not previously associated with such resistance in *An. funestus*. A particular transcript (Afun07369) had a close hit to *CYP6P9a*, but none of the common probes for this gene highly overexpressed in southern Africa was observed in Uganda. CYP4 subfamily genes overexpressed included *CYP4H17*, *CYP4C36*, and *CYP4K2*, whereas *CYP9J11*, from the CYP9 subfamily, was also overexpressed in both comparisons. Glutathione S-transferases (GSTs) were also significantly overexpressed in pyrethroid resistant mosquitoes from Uganda compared to the susceptible FANG strain, notably genes of the epsilon class, including *GSTe1* (Afun013481) (FC6.6 and 5.6, respectively, in R-S and C-S), *GSTe5* (Afun009866) and *GSTe2* (Afun000045), which, with FC of 2.9 and 2.1, exhibits a lower FC than the level observed in West Africa (Riveron *et al.* 2014b). GST genes from the Delta class were also overexpressed, including *GSTD3* (Afun008354) (FC6.6 and 5.1) and *GSTD1-5* (Afun007499). Other overexpressed detoxification gene families included sulfotransferases (with Afun013870 having a high FC of 11.9 and 14.8), carboxylesterases, aldehyde oxidases, ABC transporters, and other genes commonly associated with metabolic resistance to pyrethroid (Table 1). Cuticular protein genes were also among the overexpressed genes.

### Transcription profiling of the pyrethroid resistant population of Benin

A similar approach was used in Benin using the 4 × 44k chip, as was carried out before the design of the 8 × 60k. High numbers of probes were significantly differentially expressed for the R-S (5617) and C-S (7735) comparisons (Figure 1B), most likely due to extensive genetic differences between the samples. Contrary to Uganda, the R-C comparison also showed a high number of probes differentially expressed (6033), leading to a higher number of probes commonly differentially expressed in all three comparisons (1890).

#### R-S/C-S/R-C:

The GST *GSTe2* (Combined_c920) was the only detoxification gene commonly overexpressed in R-C, R-S, and C-S (Table 2). Three probes from this gene consistently had a higher overexpression in the R-S comparison from permethrin surviving mosquitoes *vs.* susceptible FANG than in the C-S comparison, supporting its association with permethrin resistance, in addition to its role as a main DDT metabolizer as previously established (Riveron *et al.* 2014b).

**Table 2:** List of top detoxification genes significantly overexpressed in pyrethroid resistant *An. funestus* in Benin for all comparisons

Probes	Transcript	C-S	R-C	R-S	Description
CUST_1822_PI406199769	Combined_c920	11.9	2.6	35.5	Glutathione-s-transferase gst
CUST_1822_PI406199769	Combined_c920	8.8	2.0	25.2	Glutathione-s-transferase gst
CUST_30_PI406199775	Cyp6p9b	3.9		2.9	Cytochrome p450
CUST_25_PI406199775	Cyp6p9a	6.4		2.8	Cytochrome p450
CUST_1616_PI406199772	Ee589516.1	2.3		2.6	D7-related 1 protein
CUST_8241_PI406199769	Combined_c4173	11.6		9.5	Glycoprotein 93
CUST_1964_PI406199772	Cd664227.1		2.4	2.0	Alcohol dehydrogenase
CUST_2550_PI406199769	Combined_c1287		2.4	2.3	Aldehyde dehydrogenase
CUST_3110_PI406199772	Cd577844.1		4.8	4.7	Cuticle protein
CUST_13273_PI406199769	Combined_c6791 (CYP9J11)		2.6	2.5	Cytochrome p450
CUST_13272_PI406199769	Combined_c6791 (CYP9J11)		2.8	2.6	Cytochrome p450
CUST_604_PI406199772	Ee589610.1		3.2	2.8	D7-related 1 protein
CUST_1090_PI406199798	Agap000881-ra			2.1	Aldehyde dehydrogenase
CUST_2068_PI406199798	Agap002182-ra			2.7	ABC transporter
CUST_4410_PI406201128	Agap001777-ra			3.0	ABC transporter
CUST_3109_PI406199772	Cd577844.1			5.0	Cuticle protein
CUST_4919_PI406199772	Bu038983			4.7	Cuticle protein
CUST_3398_PI406199772	Cd577693.1			5.2	Cuticle protein
CUST_48_PI406199775	Cyp6z3			2.5	Cytochrome p450
CUST_10_PI406199775	Cyp6p1			2.1	Cytochrome p450
CUST_43_PI406199775	Cyp6z1			2.4	Cytochrome p450
CUST_45_PI406199775	Cyp6z1			3.3	Cytochrome p450
CUST_27_PI406199775	Cyp6p9a			2.6	Cytochrome p450
CUST_44_PI406199775	Cyp6z1			3.5	Cytochrome p450
CUST_717_PI406199772	Ee589504.1			9.0	D7-related 1 protein
CUST_359_PI406199772	Ee589855.1			9.1	D7-related 1 protein
CUST_1687_PI406199772	Ee589439.1			8.2	D7-related 1 protein
CUST_959_PI406199772	Ee589285.1			3.6	Gsg6 salivary protein
CUST_379_PI406199772	Ee589823.1			2.2	Gsg7 salivary protein
CUST_5934_PI406199769	Combined_c3002			2.4	Superoxide dismutase
CUST_21644_PI406201128	Agap006867-ra	5.2			Adult-specific cuticular protein acp-20
CUST_120_PI406199788	Gb-COEAE1G	5.1			Alpha-esterase
CUST_21714_PI406201128	Agap010906-ra	3.6			Cuticle protein
CUST_2401_PI406199769	Combined_c1211	2.1			Glucosyl glucuronosyl transferases
CUST_178_PI406199772	Ee590018.1	2.2			Gsg7 salivary protein
CUST_6_PI406199775	Cyp6aa1		2.0		Cytochrome p450
CUST_11_PI406199775	Cyp6p1		2.5		Cytochrome p450
CUST_24_PI406199775	Cyp6p5		2.1		Cytochrome p450
CUST_26_PI406199775	Cyp6p9a		2.2		Cytochrome p450
CUST_1682_PI406199772	Ee589442.1		2.5		D7 protein
CUST_1182_PI406199772	Ee589982.1		2.5		D7-related 1 protein
CUST_892_PI406199772	Ee589340.1		2.8		D7-related 3 protein
CUST_3946_PI406199772	Cd577403.1		3.2		Glutathione s-transferase
CUST_14377_PI406199769	Combined_c7513		2.6		Glutathione transferase

#### Common probes between two comparisons:

Among probes significantly overexpressed in at least two comparisons, the cytochrome P450 genes *CYP6P9a* and *CYP6P9b* were upregulated in both C-S and R-S, but with relatively low levels compared to previously reported levels in southern Africa (<6.4 FC). Two probes of the *CYP9J11* were also upregulated, but between R-C and R-S only (Table 2). Other detoxification genes were upregulated, but only in one comparison. Those found in R-S included only the cytochrome P450s *CYP6Z1* (three probes), *CYP6Z3*, *CYP6P1*, and another probe for *CYP6P9a*. It also included two ABC transporter genes (probes from *An. gambiae* transcripts AGAP002182 and AGAP001777, respectively), an aldehyde dehydrogenase, and cuticular protein genes (Table 2). Genes only present in the C-S comparison included an alpha-esterase (COEAE1G; FC5.1) and an UDP glycosyl transferase. Other detoxification genes, including the cytochrome P450s *CYP6AA1*, *CYP6P5*, and two GSTs, were upregulated only in the R-C comparison (Table 2).

### GO enrichment analysis

Blast2go enrichment analysis for the set of probes upregulated in R-S and C-S comparisons did not detect many GO terms related to detoxification process in mosquitoes. In the case of the C-S comparison in Benin, for example, the major GO terms over-represented belong mainly to serine-type endopeptidase activity, odorant binding activity, protein DNA complex, and others (Figure S1). Similar results were obtained for other comparisons. The lack of GO terms associated with detoxification is similar to previous studies with this microarray chip in *An. funestus* (Riveron *et al.* 2013, 2014a). This is probably caused by the poor annotation of the set of Expressed Sequences Tags (ESTs) used for the microarray chip, and the composite nature of the microarray chip made of transcripts from different sources.

### Regional comparison of expression profiles between West (Benin), East (Uganda) and southern (Malawi) Africa

The variation in the underlying resistance mechanisms to pyrethroid between geographical regions in Africa was analyzed by comparing the expression profiles from Benin and Uganda to that from Malawi in southern Africa using the 8 × 60k chip. The number of significantly differentially expressed probes is presented in Figure 1C.

#### Genes common in all regions:

Among the genes the most upregulated in all three regions were a trypsin-related protease gene (Afun008293), the P450 *CYP6M7*, the argininosuccinate lyase, and a glycogenin gene. However, although overexpressed in all regions, the expression levels vary significantly for some genes, such as for *CYP6M7*, which has a FC of 131.9 in Benin but only 12.5 in Malawi (Table 3). Among the detoxification genes commonly overexpressed in all three regions, cytochrome P450s were again dominant. Most of these P450 genes showed a similar level of expression in all the three countries, and included *CYP4H17*, *CYP6Z1*, *CYP6M4*, *CYP6M2*, *CYP9J11*, *CYP9J13*, *CYP304B1*, and a gene close to *CYP6P9a* (Afun007369). Another P450, *CYP9K1*, although commonly overexpressed in all three countries, was significantly more highly present in Uganda (FC16.1) than in Malawi (FC2.4) and Benin (FC6.2), suggesting a bigger role for this gene in Uganda. Other commonly expressed detoxification genes included an aldehyde oxidase (AGAP006220) and a UDP glucuronosyl transferase (AGAP006222).

**Table 3:** Detoxification genes commonly upregulated in Uganda (UG), Malawi (MAL), and Benin (BN) countries

Probe Name	*An. funestus* ID	*An. gambiae* ID	UG	MAL	BN	Description
CUST_8293_PI426302897	Afun008293	AGAP008291-PA	83.0	32.5	74.4	Trypsin-related protease
CUST_7663_PI426302897	Afun007663 (CYP6M7)	AGAP008213-PA	24.0	12.5	131.9	Cytochrome p450
CUST_500_PI426302897	Afun000500		23.0	8.8	17.1	Glycogenin
CUST_9227_PI426302897	Afun009227	AGAP008141-PA	21.2	66.3	29.2	Argininosuccinate lyase
CUST_8887_PI426302897	Afun008887	AGAP011997-PA	17.6	8.5	7.3	Nucleotide binding protein 2 (nbp 2)
CUST_7769_PI426302897	Afun007769 (CYP9K1)	AGAP000818-PA	16.1	2.4	6.2	Cytochrome p450
CUST_1392_PI426302897	Afun001392		10.7	8.8	6.0	Glycine dehydrogenase
CUST_11042_PI426302897	Afun011042	AGAP003321-PA	8.1	18.5	6.4	Glycine dehydrogenase
CUST_4223_PI426302897	Afun004223 (CYP4H17)	AGAP008358-PA	7.5	6.8	9.5	Cytochrome p450
CUST_10949_PI426302897	Afun010949	AGAP010887-PA	7.4	3.0	4.2	Cuticular protein rr-1 family
CUST_1459_PI406199769	combined_c738		7.3	6.4	10.9	Short-chain dehydrogenase
CUST_6930_PI426302897	Afun006930 (CYP6M2)	AGAP008212-PA	5.3	4.3	5.0	Cytochrome p450
CUST_3246_PI426302897	Afun003246	AGAP006220-PA	4.5	3.8	4.2	Aldehyde oxidase
CUST_1563_PI406199772	EE589574.1		4.4	2.1	2.8	D7-related 1 protein
CUST_12343_PI426302897	Afun012343 (CYP4H17)	AGAP008358-PA	4.4	2.9	4.2	Cytochrome p450 4d1
CUST_8347_PI426302897	Afun008347	AGAP009828-PA	3.9	4.2	5.0	Chymotrypsin 1
CUST_9522_PI426302897	Afun009522 (CYP9J13)	AGAP012292-PA	3.5	4.5	2.8	Cytochrome p450
CUST_1710_PI406199772	EE589412.1		3.3	2.2	2.6	D7-related 1 protein
CUST_12197_PI426302897	Afun012197 (CYP304B1)	AGAP003066-PA	3.3	2.8	4.1	Cytochrome p450
CUST_10360_PI426302897	Afun010360	AGAP006222-PA	3.2	2.0	4.8	Glucosyl glucuronosyl transferases
CUST_9584_PI426302897	Afun009584 (CYP6M4)	AGAP008214-PA	3.2	3.2	3.3	Cytochrome p450
CUST_27_PI426302915	CYP6Z1		3.1	2.5	2.3	Cytochrome p450
CUST_198_PI406199772	EE590001.1		3.0	2.1	3.1	D7-related 1 protein
CUST_7369_PI426302897	Afun007369 (CYP6P9a-like)	AGAP002865-PA	3.0	2.5	4.4	Cytochrome p450
CUST_7469_PI426302897	Afun007469 (CYP9J11)	AGAP012296-PA	3.0	3.1	2.7	Cytochrome p450
CUST_3109_PI406199772	CD577844.1		2.9	2.5	2.4	Cuticle protein
CUST_9335_PI426302897	Afun009335	AGAP003343-PA	2.8	2.7	2.7	Cytochrome p450
CUST_2473_PI426302897	Afun002473	AGAP000553-PA	2.5	4.5	2.5	ATP-binding-cassette protein
CUST_7861_PI426302897	Afun007861	AGAP008219-PA	2.5	3.1	2.2	Cytochrome p450
CUST_1097_PI406199769	Combined_c557		2.5	6.4	5.1	Trypsin
CUST_10_PI426302915	CYP6M4.seq		2.4	2.6	3.2	Cytochrome p450
CUST_798_PI426302897	Afun000798 (CYP6M2)	AGAP008212-PA	2.1	2.5	2.6	Cytochrome p450

#### Genes common in only two regions:

Analysis of the list of genes commonly overexpressed in only two regions revealed that, for Uganda and Benin, the GSTs *GSTe1* and *GSTd3* were common to both countries, as were the P450s *CYP307A*, *CYP314A1*, and *CYP315A*. For those overexpressed only in Uganda and Malawi, the P450 *CYP4C27* was detected, although with a higher expression in Uganda (FC10.3) than in Malawi (FC2.1). The *CYP4C36* was also upregulated in both countries, similar to *GSTd1-5*. Other genes are also listed in Table S2 in File S1. The list of genes overexpressed only in Malawi and Benin is dominated by the *CYP6P9a* and *CYP6P9b* with several probes, but with a far higher overexpression in Malawi for both genes (*e.g.*, FC39.4 for *CYP6P9a* in Malawi *vs.* only FC4.3 in Benin), suggesting that both genes are mainly driving resistance in southern Africa.

Some genes common to all countries were detected through different probes, such as *GSTe2*, which in Benin and Malawi was detected by probes against Combined_c920 transcript, whereas in Uganda and Malawi it was through probes for Afun000045 transcript, showing the impact of sequence polymorphism in the microarray results.

#### Quantitative RT-PCR:

Key genes exhibiting striking differences between regions (*CYP6P9a*, *CYP6P9b*, and *GSTe2*) or commonly overexpressed in all three countries (*CYP9J11*) were further validated by qRT-PCR. Analysis of the expression patterns confirmed the differences observed with microarrays, as both *CYP6P9a* and *CYP6P9b* were highly overexpressed only in Malawi, but just barely in Uganda and Benin, as seen with microarray (Figure 1D). Similarly, the *GSTe2* was highly overexpressed in Benin permethrin resistant individuals (FC 73.1), but only very low-level expression of this gene was observed in Uganda and Malawi. The common overexpression of the *CYP9J11* was also validated in all three countries, although at a lower fold-change compared to the other genes.

### Functional characterization of key genes commonly overexpressed in all countries

Several genes (notably P450s) commonly overexpressed in the three geographical regions are located in the chromosomal regions spanning the three QTL (rp1, rp2, and rp3) previously detected for pyrethroid resistance in *An. funestus* (Wondji *et al.* 2009). Although the key genes driving resistance in rp1 and rp2 have already been characterized (Irving *et al.* 2012; Riveron *et al.* 2013), the genes driving resistance in rp3 remain uncharacterized. The *CYP9J11* overexpressed in all three regions, and located in the 3L chromosomal region spanning the rp3 QTL, could be the pyrethroid metabolizer gene in this QTL. To validate this hypothesis, we performed a functional characterization of this gene.

#### Polymorphism analysis of CYP9J11:

**Figure 2:**
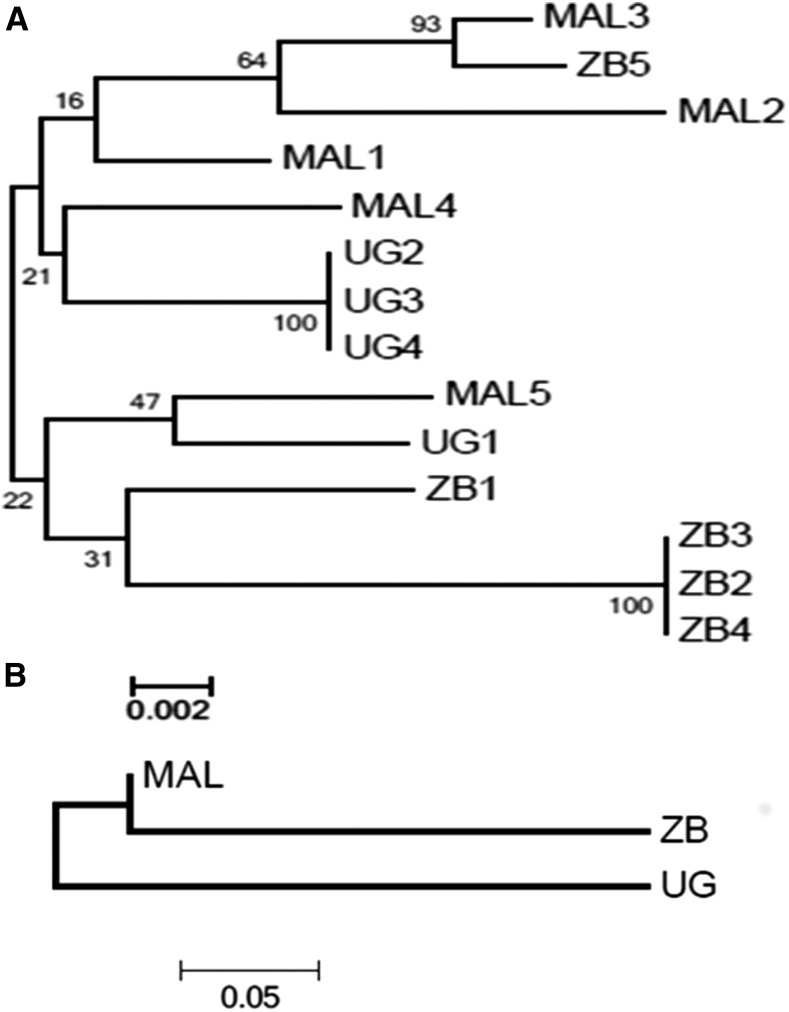
Genetic diversity pattern of CYP9J11 in East (Uganda; UG) and southern [Malawi (MAL) and Zambia (ZB)] Africa. (A) Maximum likelihood tree of *CYP9J11* from the cDNA haplotypes of the full-length 1644 bp sequence. (B) Genetic distances between African populations (*Nst* estimates) between the three countries.

Analysis of the genetic variability of *CYP9J11* full-length cDNA (1644 bp) for five clones each from Malawi and Zambia, and four from Uganda revealed a high polymorphism of this gene, with an average of 93 polymorphic sites observed for all combined 14 sequences and 17 amino acid changes observed in total (Table S3 in File S1). No evidence of directional selection was detected on *CYP9J11*, as shown by the lack of significant Tajima D and Fu and Li D’ estimates. No specific clades per the location of origin was observed between haplotypes, although the genetic distance tree revealed a closer genetic similarity between Malawi and Zambia than Uganda, as expected from geographical distance (Figure 2, A and B).

### Functional validation of CYP9J11 using heterologous expression in E. coli and metabolism assays

#### Pattern of expression of CYP9J11:

On average, CYP9J11 protein consistently expressed at low concentration (0.13 ± 0.007 nmol/mg protein) compared with previous estimates reported for CYP6M7 (0.15 ± 0.0 nmol/mg protein), and for CYP6P9a (0.42–1.0 nmol/mg) and CYP6P9b (0.35–0.42 nmol/mg), respectively.

#### Assessment of CYP9J11 pyrethroid activities and cross-resistance using metabolism assays:

**Figure 3:**
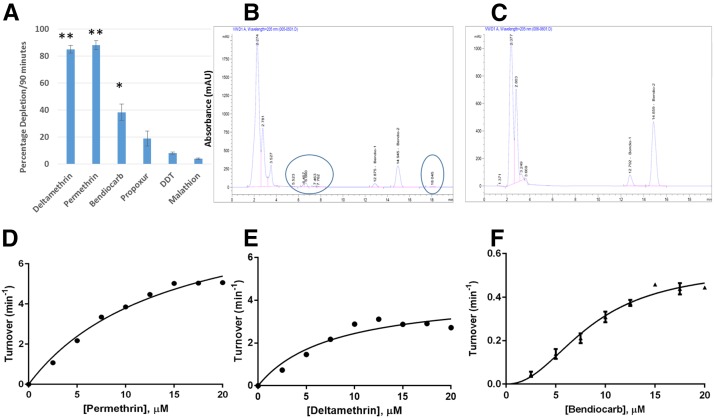
Functional validation of the role of CYP9J11 P450 gene in carbamate/pyrethroid resistance. (A) Percentage depletion of 20 µM carbamate and pyrethroid insecticides with *CYP9J11*. Results are an average of three replicates (*n* = 3) compared with negative control. * and ** Significantly different from negative control (−NADPH) at *P* < 0.05 and *P* < 0.01, respectively. (B) Polar metabolites with short retention time eluted at the beginning of chromatogram of CYP9J11 metabolism of bendiocarb (NADPH+). A third putative metabolite of bendiocarb metabolism eluted at 18.045 min. (C) Chromatogram of NADPH- incubation tubes devoid of polar metabolites with short retention indicating no metabolism of bendiocarb in the absence of NADPH regeneration agent. (D, E) Michaelis-Menten plot of CYP9J11 mediated metabolism of permethrin and deltamethrin, respectively. Results are an average of three replicates (*n* = 3) compared with negative control; (F) Allosteric sigmoidal curve of CYP9J11 metabolism of bendiocarb. Results are average of three replicates (*n* = 3) compared with negative control. K_half_ = *K*_M_; *h* = 2.29.

Disappearance of 20 µM insecticide substrates was determined after 90 min of incubation with the recombinant CYP9J11 in the presence of cytochrome b_5_ and NADPH regeneration system. CYP9J11 metabolized permethrin and deltamethrin with significant depletions of 88.05% ± 3.23 (*P* < 0.0001) and 95.05% + 0.74 (*P* < 0.0001), respectively (Figure 3A). These depletions were higher than obtained with both *CYP6P9a* and *CYP6P9b* alleles (Riveron *et al.* 2013, 2014a). Carbamates bendiocarb and propoxur, as well as the organophosphate malathion, were screened to investigate potential cross resistance by CYP9J11. Low and nonsignificant depletion was observed against DDT and malathion (Figure 3A), as observed previously from CYP6P9a, CYP6P9b, and CYP6M7. This result is consistent with malathion susceptibility across Africa so far. CYP9J11 significantly depleted bendiocarb; but with a lower depletion of 38.34% ± 7.01 (*P* < 0.05) than previously reported for CYP6Z1 (54.72% ± 0.45, *P* < 0.05) (Ibrahim *et al.* 2016a). In contrast to incubations with CYP6M7, CYP6P9a, and CYP6P9b (<10% depletions), CYPJ11-mediated metabolism of bendiocarb proceeded with polar metabolites eluting in the beginning of the HPLC chromatogram (Figure 3B). Initial reaction of carbamate metabolism has been described to produce very polar products that remain at the origin of the chromatogram (Kuhr 1970) and such highly polar metabolites have been recently described in metabolisms assay with bendiocarb and *An. funestus* CYP6Z1 protein (Ibrahim *et al.* 2016a).

#### Kinetics parameters of CYP9J11 metabolism of insecticides:

The CYP9J11-mediated metabolism of permethrin and deltamethrin follows a Michaelis-Menten pattern (Figure 3, A and B), but a decline in activity was observed with deltamethrin above 12.5 µM concentration, attributed to substrate or product inhibition. The turnover (*K*_cat_) and *K*_M_ obtained with permethrin was 9.260 min^−1^ ± 1.048 and 14.39 µM ± 3.12 leading to a catalytic efficiency of 0.643 min^−1^ µM^−1^ ± 0.157. The turnover for deltamethrin (4.338 min^−1^ ± 1.381) was, on average, half the value obtained with permethrin, but the affinity of CYP9J11 toward deltamethrin was surprising higher (*K*_M_ of 7.957 ± 1.31). The catalytic efficiency of CYP9J11 for deltamethrin was calculated as 0.545 min^−1^ µM^−1^ ± 0.195, lower than obtained with permethrin. The catalytic efficiency of this enzyme toward permethrin is higher than obtained from *An. funestus* pyrethroid metabolizers *CYP6P9a* and *CYP6P9b* (Riveron *et al.* 2014a).

*CYP9J11* was also tested with 20 µM bendiocarb and was shown to behave in allosteric fashion with this carbamate insecticide, with positive cooperativity (Hill coefficient, *h* = 2.29 ± 0.38) as described to be the case of some P450s (Atkins 2004). *CYP9J11* portrayed sigmoidal curve with relatively low *K*_half_ (lower than *K*_M_ obtained with pyrethroids) and low maximal catalytic rate (Figure 3C). The dose-response curve was thus modeled using the GraphPad prism with relevant module as described (Copeland 2004). The V_max_ and K_half_ (*K*_M_) for bendiocarb were calculated as 0.04 min^−1^ ± 0.005 and 0.75 µM ± 0.2, respectively, leading to a very low catalytic efficiency of 0.053 min^−1^ µM^−1^ ± 0.0157, 12 times lower than compared with the values obtained with permethrin.

### Transgenic expression of candidate genes in Drosophila flies

#### Validation of role of CYP9J11:

**Figure 4:**
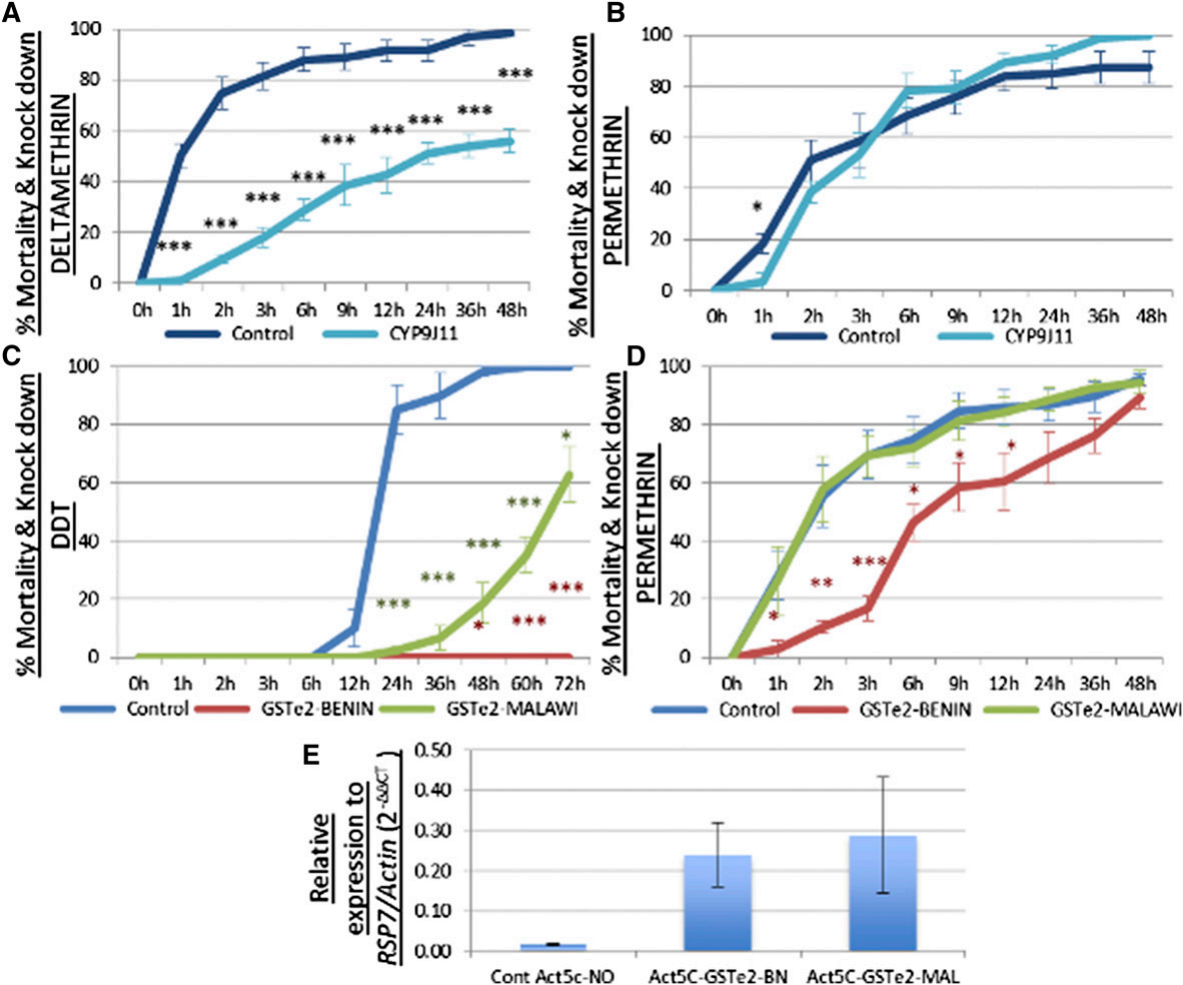
Functional validation of the role candidate resistance genes using transgenic expression in flies: (A) results of bioassay analysis of transgenic flies overexpressing *CYP9J11* Act5C-CYP9J11 *vs.* control flies for deltamethrin. (B) The same bioassays with permethrin. (C) Functional validation of the role of allelic variation at the *GSTe2* genes on the resistance phenotype using transgenic expression in flies through a comparative transgenic analysis of the 119F and L119-GSTe2 alleles using bioassay tests on transgenic Act5C-GSTe2-119F (GSTe2-Benin) and Act5C-GSTe2-L119 (GSTe2-Malawi) and flies (Exp-GSTe2), control strains [two parental (UAS-GSTe2 and GAL4-Actin) and F_1_ progeny that do not express the *GSTe2* transgene (Cont-NO)]. (D) The same bioassays with permethrin. (E) Relative expression of the transgene *GSTe2* alleles in the transgenic *D. melanogaster* strain (Act5C-GSTe2-MAL and Act5C-GSTe2-BN) and the control sample with no *GSTe2* expression (Cont Act5c-NO). Data shown as mean ± SEM significantly different: *P < 0.05, **P < 0.01, and ***P < 0.001.

To confirm that *CYP9J11* overtranscription alone can confer pyrethroid resistance, transgenic *D. melanogaster* individuals were generated expressing *CYP9J11* (derived from permethrin resistant field mosquitoes from Uganda) under the control of the ubiquitous Act5C-GAL4 driver. Contact bioassays performed with 2% permethrin (type I pyrethroid) and 0.15% deltamethrin (type II) revealed that *CYP9J11* overtranscription alone is sufficient to confer resistance to this class of insecticide. For deltamethrin, the flies overexpressing *CYP9J11* were resistant with a significantly reduced mortality/knockdown rate compared to that observed for control flies (Figure 4A). Significantly reduced mortality/knockdown rates were recorded at all nine different exposure times for transgenic Act5C-CYP9J11 individuals when compared with the control group not expressing *CYP9J11*. For example, mortality rates were 1.04 ± 1% *vs.* 50.3 ± 4.4% (*P* < 0.001) at 1 hr, 9.5 ± 1.7% *vs.* 74.7 ± 6.32% (*P* < 0.001) at 2 hr and 56.03 ± 4.6% *vs.* 98.3 ± 3.3% (*P* < 0.001) at 24 hr (Figure 4A). These results demonstrate that *CYP9J11* overtranscription alone is sufficient to confer resistance to deltamethrin. For permethrin, significantly reduced mortality/knockdown rate was recorded for transgenic Act5C-CYP9J11 flies when compared with the control after 1 hr exposure (3.33 ± 3.3% *vs.* 18.36 ± 3.8%; *P* < 0.05). However, similar mortality rates were recorded for both experimental and control samples at the rest of the exposure times, with no significant differences observed (Figure 4B).

#### Confirmation of role of allelic variation of GSTe2 in both DDT and pyrethroid resistance:

Due to the consistent overexpression of *GSTe2* in permethrin-resistant mosquitoes in Benin where the L119F mutation is fixed, the role of the allelic variation on this gene was investigated using transgenic expression. Comparative bioassays performed between a transgenic line expressing the susceptible L119 allele, and another one expressing the 119F resistant allele, revealed that the 119F mutation confers a higher resistance against both DDT and permethrin. For DDT, no mortality is observed in the flies expressing the resistant 119F allele for all the different exposure times, whereas significantly higher mortality rates were observed for the flies expressing the susceptible L119 allele from 24 to 72 hr exposure time (2.2–63%; *P* < 0.001) (Figure 4C). However, the fact that these mortality rates for flies expressing the susceptible L119 allele were lower than for flies not expressing the *GSTe2* (2.22 ± 1.4, 18.65 ± 7.1, and 62.69 ± 9.6% *vs.* 85.12 ± 8.4, 98.33 ± 1.7 and 100%; *P* < 0.001; respectively at 24, 48, and 72 hr) suggests that even overexpression of the susceptible allele provide resistance against DDT in flies, but at a significantly lower level than with the 119F resistance allele. Bioassays with permethrin revealed that only flies expressing the resistant 119F allele had significantly lower mortality rate compared to control flies not expressing *GSTe2* (2.78 ± 2.7, 10.52 ± 2.1, 16.74 ± 4.3, and 46.40 ± 6.07% *vs.* 27.98 ± 8.3, 55.44 ± 10.4, 69.60 ± 8.4, and 74.81 ± 7.8%; *P* < 0.01; respectively at 1, 2, 3, and 6 hr exposure time) (Figure 4D). Flies expressing the susceptible L119 allele showed the same high mortality rates as the control flies. Each GSTe2 allele was confirmed to be expressed only in the F_1_ progeny of the GAL4/UAS crosses by qRT-PCR (Figure 4E). 

### CYP6P9a and CYP6P9b polymorphisms in Uganda in comparison to southern Africa

**Figure 5:**
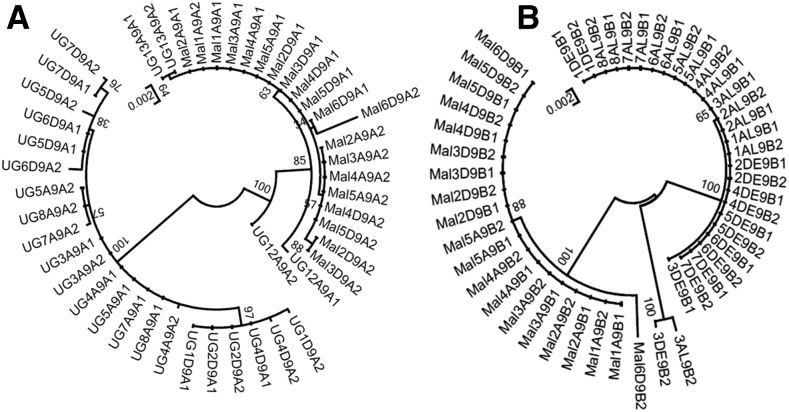
Molecular phylogenetic analysis of *CYP6P9a* (A) and *CYP6P9b* (B) in Uganda (UG) for both permethrin resistant and susceptible mosquitoes in comparison to Malawi (Mal) using the Maximum Likelihood method. The evolutionary history was inferred by using the Maximum Likelihood method based on the Tamura 3-parameter model. The tree with the highest log likelihood is shown. Initial tree(s) for the heuristic search were obtained by applying the Neighbor-Joining method to a matrix of pairwise distances estimated using the Maximum Composite Likelihood (MCL) approach. The tree is drawn to scale, with branch lengths measured in the number of substitutions per site. The analysis involved 46 (*CYP6P9a*) and 50 (*CYP6P9b*) nucleotide sequences. All positions containing gaps and missing data were eliminated. There were a total of 1990 (*CYP6P9a*) and1757 (*CYP6P9b*) positions in the final dataset. Evolutionary analyses were conducted in MEGA6.

A comparative analysis of the polymorphism pattern of the duplicated P450 genes *CYP6P9a* and *CYP6P9b* was performed between permethrin-resistant and -susceptible mosquitoes from Uganda and those from Malawi. The aim was to assess whether the low expression of these genes in Uganda correlated with a higher genetic diversity of both genes in contrast to southern African, where a high overexpression was associated with a directional selection with reduced genetic diversity (Riveron *et al.* 2013, 2014a). Overall, both *CYP6P9a* and *CYP6P9b* genes exhibit a higher genetic diversity in Uganda than in Malawi as shown by the number of substitutions (50 *vs.* 13 for *CYP6P9a*; 45 *vs.* 12 for *CYP6P9b*), haplotypes (15 *vs.* 5 for *CYP6P9a*; 4 *vs.* 2 for *CYP6P9b*), and estimates of genetic diversity or number of nonsynonymous substitutions (Table S3 in File S1). This higher genetic diversity of both genes in Uganda correlates with their low overexpression and support a lower role of both genes in Uganda. However, the maximum likelihood trees of haplotypes of both genes for Uganda and Malawi (Figure 5, A and B) revealed that for *CYP6P9a*, four haplotypes from resistant mosquitoes clustered with haplotypes from Malawi. These Uganda haplotypes also exhibit the insertion of two AAs [CAAAAAA(AA)] in the promoter region characteristic of southern African resistant haplotypes (Ibrahim *et al.* 2015). For both genes, haplotypes of both countries cluster in separate clades (Figure 5, A and B).

## Discussion

Elucidation of resistance mechanisms to insecticide in mosquito vectors of tropical diseases such as malaria is a prerequisite for a better management of the growing problem of resistance to existing insecticide classes in public health sectors. If progress has been made in assessing the local transcription profiles associated with pyrethroid resistance in malaria vectors in Africa, generating a broader view of the molecular basis of resistance continent-wide has been limited. The regional comparison of the transcription profile of pyrethroid resistance in *An. funestus* across Africa revealed three main lessons discussed below.

### The transcription profile of pyrethroid resistance is not uniform across the continent

The genome-wide analysis of the transcription profile associated with pyrethroid resistance highlighted a common trait, *i.e.*, the predominant role of cytochrome P450 genes in the metabolic resistance observed in *An. funestus* population as previously reported in southern Africa (Riveron *et al.* 2013, 2014a) and in other mosquito species such as *An. gambiae* (Mitchell *et al.* 2012; Kwiatkowska *et al.* 2013) or in *Aedes* (Strode *et al.* 2008; Bariami *et al.* 2012; Saavedra-Rodriguez *et al.* 2012; Ishak *et al.* 2016). However, the drastic difference in the expression levels of key P450s suggests that the origin of resistance is not the same across the continent, and that there were independent selection events of resistance to pyrethroids in various populations. A clear example is that provided by the expression profile of the duplicated P450s *CYP6P9a* and *CYP6P9b*, the main pyrethroid resistance genes in southern African populations of *An. funestus* (Amenya *et al.* 2008; Riveron *et al.* 2013, 2014a), but which, from this study, seem to play no or little role in East Africa, as further supported by their higher genetic diversity in Uganda than in Malawi but also than in West (Benin and Ghana) and Central Africa (Cameroon) (Barnes *et al.* 2017). Such drastic variation is also in line with the gradual reduced expression of *CYP6P9a* and *CYP6P9b* in Zambia compared to Malawi and Mozambique (Riveron *et al.* 2014a; Thomsen *et al.* 2014; Barnes *et al.* 2016), and suggests barriers to gene flow previously detected between African populations of this mosquito species (Michel *et al.* 2005; Barnes *et al.* 2017). Variation in the transcription profiles of insecticide resistance genes are also reported in other mosquito species such as *An. gambiae*, where P450 genes such as *CYP6P3* and *CYP6M2* highly overexpressed in West (Mitchell *et al.* 2012; Kwiatkowska *et al.* 2013) and in Central (Fossog Tene *et al.* 2013; Antonio-Nkondjio *et al.* 2016) Africa are not significantly expressed in the southern populations in Zambia (Thomsen *et al.* 2014). Equally, in contrast, the P450 *CYP6P4* from *An. arabiensis* has also been shown to be a major driver of pyrethroid resistance in populations from Chad (Ibrahim *et al.* 2016b) and Sudan (Abdalla *et al.* 2014). It is therefore important to avoid generalizing the underlying molecular basis of resistance across countries or the continent, but rather to determine as much as possible the main resistance genes in the different countries/regions, efforts that can impact the design of diagnostic tools or resistance management strategies. For example, the *CYP6M2* in *An. gambiae* (Edi *et al.* 2014) and the *CYP6Z1* (Ibrahim *et al.* 2016a) in *An. funestus* have been shown to confer cross-resistance between pyrethroids and carbamates, so their significant overexpression in a population should prevent using carbamates as alternative to pyrethroids in an IRS campaign.

### The cytochrome P450 CYP9J11 is a common African pyrethroid resistance gene

If significant differences are observed between regions, there are also similarities with common genes observed across the continent such as the P450 *CYP9J11* which was overexpressed in all three regions assessed here. However, because of its moderate level of expression, *CYP9J11* may not be the primary resistance gene. Nevertheless, its significant catalytic efficiency in metabolising pyrethroid means it cannot be disregarded. *CYP9J11* is the ortholog of *CYP9J5* in *An. gambiae*, which was recently shown to also metabolize pyrethroids and pyriproxyfen, and to be overexpressed Africa-wide in *An. gambiae* field populations from West (Toe *et al.* 2015), Central (Fossog Tene *et al.* 2013), and East Africa (Nkya *et al.* 2014), suggesting that this gene could be important in providing protection to a wide range of xenobiotics in malaria vectors. *CYP9J11* is also located on the 3L chromosome where the *rp3* (resistance to pyrethroid 3) QTL had previously been detected, suggesting that it could be the main gene behind *rp3* (Wondji *et al.* 2005, 2007, 2009). In addition to the ability to metabolize pyrethroids and confer resistance to *An. funestus*, *CYP9J11* as previously shown for *CYP6Z1* (Ibrahim *et al.* 2016a) is a cross-resistance gene, able to metabolize nonpyrethroid insecticides used in public health using noncanonical Michaelis-Menten kinetic mechanisms. Various P450s exhibit functional allostery using distributive catalysis to minimize toxicological effects of substrates (Atkins *et al.* 2002), for example, the promiscuous *CYP3A4* (Wang *et al.* 2000), *CYP2C9* (Tracy *et al.* 2002), and the recently characterized *An. funestus CYP6Z1* (Ibrahim *et al.* 2016a). At low substrate concentrations, the slower substrate turnover afforded by cooperative CYPs compared with Michaelis-Menten enzymes can be a significant toxicological advantage, when toxic thresholds exist (Atkins *et al.* 2002). Possibly, bendiocarb is too “toxic” for *CYP9J11*, even though it can metabolize it, and this is why P450 employs distributive catalysis to effect its catalysis, as in the case of *An. funestus CYP6Z1* (Ibrahim *et al.* 2016a).

### 3-Allelic variation of GST GSTe2 impacts pyrethroid resistance

The significant overexpression of *GSTe2* in Benin in pyrethroid resistant mosquitoes (as seen by the FC > 2 in R-C comparing permethrin resistant to control, not-exposed, mosquitoes from Pahou) suggested that this gene could be involved in permethrin resistance. The significant lower mortality observed in transgenic *Drosophila* flies expressing the resistant 119F allele compared to those expressing the susceptible L119 allele supports the key role that allelic variation in this gene plays beside its overtranscription. As previously shown for DDT resistance, it is likely that the 119F also enlarging the GSTe2 binding cavity to facilitate access of pyrethroid, and allow either sequestration as suggested for GST action on pyrethroids (Vontas *et al.* 2005), or a direct metabolism as established by Riveron *et al.* (2014a). The ability of the transgenic expression in *Drosophila* flies to establish the phenotypic impact of the allelic variation of *GSTe2* due to a single amino acid change highlights the robustness of this approach in functionally characterizing the role of candidate resistance genes in conferring resistance to insecticide. This shows that experimental results from transgenic *Drosophila* are very relevant to the phenotype obtained in mosquitoes, while providing the advantage that studies in *Drosophila* could be easily scaled up to hundreds of genes, with less work, cost and space required for storage of transgenic lines. Allelic variation was also recently shown to play an important role in the pyrethroid resistance conferred by the duplicated P450s *CYP6P9a* and *CYP6P9b* in southern African populations of *An. funestus* (Ibrahim *et al.* 2015), suggesting that, besides overtranscription of detoxification genes, amino acid changes in coding regions could also play a major role. Such cases will facilitate the design of DNA-based diagnostic tools to detect metabolic resistance in field populations, as done already for the L119F-GSTe2 mutation (Riveron *et al.* 2014a).

### Conclusion

The comparative transcription analysis performed in this study between various African regions highlights that, although metabolic resistance is the common driving mechanism of pyrethroid resistance in *An. funestus* populations, there are significant special variations on the main genes associated with it, which could impact patterns of cross-resistance and resistance management strategies. The impact of many genes conferring resistance and cross-resistance to multiple resistant populations of *An. funestus* in sub-Saharan African is a challenge to resistance management. This phenomenon makes the resistance highly heterogeneous and complex, making the design of appropriate diagnostic tools operationally challenging. There is an overwhelming need for newer classes of insecticides that are safe but potent enough to control mosquito vectors of malaria and other diseases effectively. But caution must be exercised because of the presence of a number of detoxification enzymes that can confer cross-resistance, and a new insecticide may already be doomed before being deployed if resistance genes can already metabolize it.

## Supplementary Material

Supplemental material is available online at www.g3journal.org/lookup/suppl/doi:10.1534/g3.117.040147/-/DC1.
