# Supplementary material for: Genome-Wide Transcription and Functional Analyses Reveal Heterogeneous Molecular Mechanisms Driving Pyrethroids Resistance in the Major Malaria Vector Anopheles funestus Across Africa
Source: G3 (Bethesda). 2017 Apr 19;7(6):1819–32. doi: 10.1534/g3.117.040147 (PMC5473761; doi:10.1534/g3.117.040147)
Supplement: Supplementary file 1 [file 1819FigureS1.pdf]

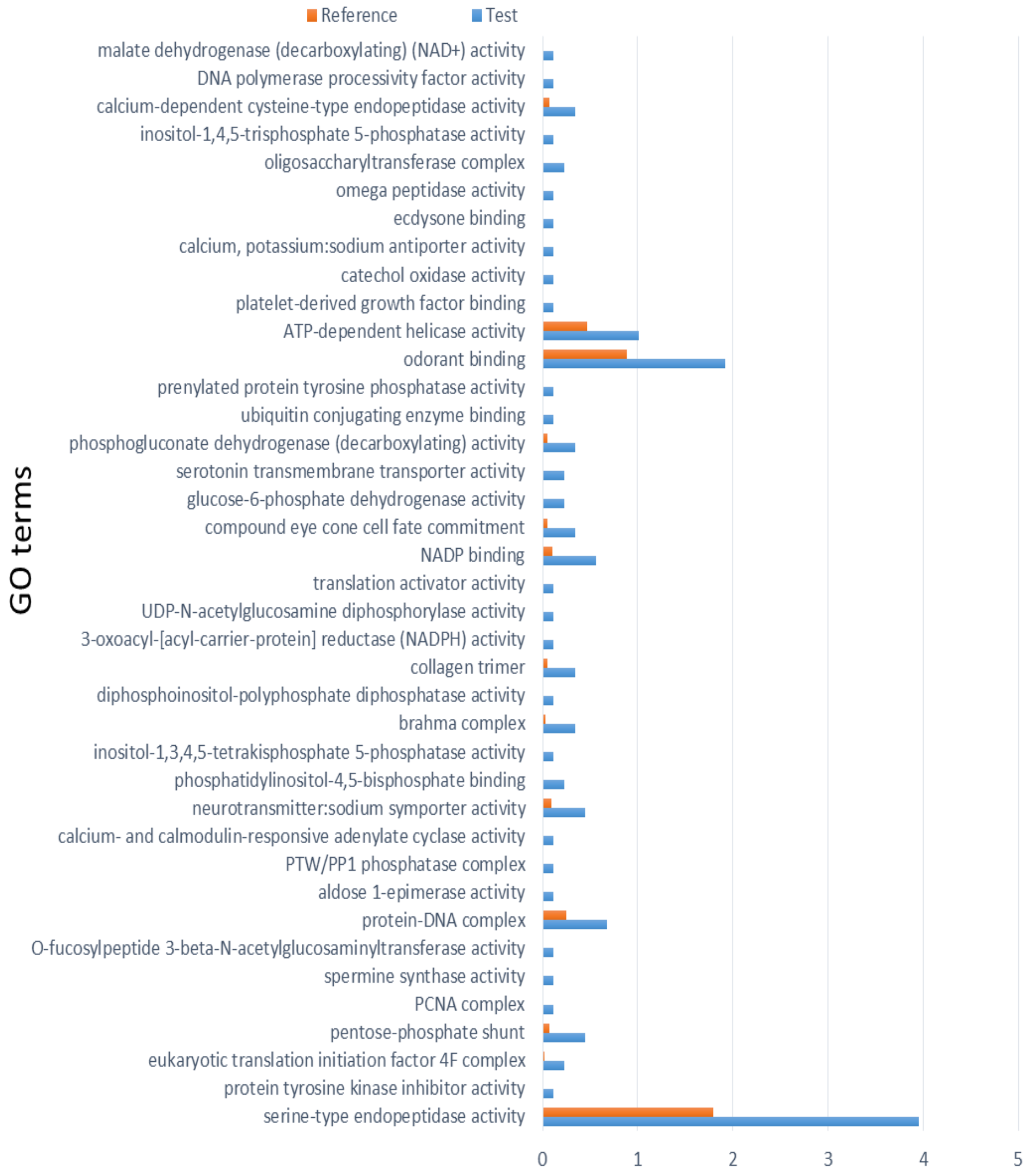

Figure S1: **Gene Ontology (GO) terms enriched in the list of probes up-regulated C-S comparisons in Benin using BLAST2GO.** The test set represents the probes over-represented while the reference set is composed of the complete set of probes on the microarray chip. The Fisher's exact test and Benjamini and Hochberg multiple testing correction was used to assess the significance of the enrichment (P< 0.05).
