# Supplementary material for: Genome-Wide Transcription and Functional Analyses Reveal Heterogeneous Molecular Mechanisms Driving Pyrethroids Resistance in the Major Malaria Vector Anopheles funestus Across Africa
Source: G3 (Bethesda). 2017 Apr 19;7(6):1819–32. doi: 10.1534/g3.117.040147 (PMC5473761; doi:10.1534/g3.117.040147)
Supplement: Supplementary file 2 [file 1819FileS1.pdf]

Table S1: List of primers used in the study

	Forward primer	Reverse primer	Expected size (bp)
**qRT-PCR primers**			
GSTe2	GTTTGAAGCAGTTGCCATACTACGAGG	TCAAGCTTTAGCATTTTCCTCCTTTTTGG C	101
CYP6P9a	CAGCGCGTACACCAGATTGTGTAA	TCA CAA TTT TTC CAC CTT CAA GTA ATT ACC CGC	92
CYP6P9b	CAGCGCGTACACCAGATTGTGTAA	TTA CAC CTT TTC TAC CTT CAA GTA ATT ACC CGC	97
CYP9J11	CAAATTTAAAGAGTGCGCTAGG	GTAGATGGTGCCAAGGATGG	115
Actin	TTAAACCCAAAAGCCAATCG	ACCGGATGCATACAGTGACA	111
**RSP7**	GTGTTCGGTTCCAAGGTGAT	TCCGAGTTCATTTCCAGCTC	98
**Primers for functional analysis**			
GSTe2	AGATCTATGACCAAGCTAGTTCTGTAC ACGCT	TCTAGATCAAGCTTTAGCATTTTCCTCCT TTTTGGC	
	GAATTCCATATGACCAAGCTAGTTCTG TACACGCT	CCGCTCGAGTCAAGCTTTAGCATTTTCC TCCTTTTTGGC	
CYP9J11			
**CYP9J11Full**	ATG GAG ATC GAT TTG ATG GTT CT	TTA CAT ACT AAC TTC GTT ATC TTT CTT TCT AGG	
**ompA+2F**	GGAATTCCATATGAAAAAGACAGCTA TCGCG		
**ompA+2CYP9J11_F**	AACCATCAAATCGATCTCCATCGGAGC GGCCTGCGCTACGGTAGCGAA		
**ompA+2CYP9J11_R**		TCTAGATCTAGATTACATACTAACTTCGTTATCTT TC	
**Primers used for amplification for polymorphism analysis**			
CYP6P9a	ATCCCTAACTATTAAAAGGCAAT	TCACAATTTTTCCACCTTCAAGTAATTACCCGC	2.2
CYP6P9b	CATACTCATAATAACTAGACGCG	TTACACCTTTTCTACCTTCAAGTAATTACCCGC	1.8

In red is restriction site for *Bgl*II; in green is for *Xba*I; blue is for *Nde*I; purple is for *Xho*I; *EcoR*I is dark brown.

Table S2: Detoxification genes commonly up-regulated only in two countries between Uganda (UG), Malawi (MAL) and Benin (BN)

Probe Name			UG	MAL	BN	Description
CUST_13921_PI426302897	Afun013921	AGAP006709-PA	49.5		27.4	chymotrypsin 1
CUST_11037_PI426302897	Afun011037	AGAP003581-PA	8.0		13.1	alcohol dehydrogenase
CUST_12461_PI426302897	Afun012461	AGAP000288-PA	6.8		10.5	alcohol dehydrogenase
CUST_6368_PI426302897	Afun006368	AGAP003785-PB	6.4		3.2	glucose dehydrogenase
CUST_15244_PI426302897	Afun015244	AGAP000820-PA	5.9		4.1	cuticular protein rr-1 family
CUST_13481_PI426302897	Afun013481 (GSTe1)	AGAP009195-PA	5.6		2.3	glutathione-s-transferase gst
CUST_8354_PI426302897	Afun008354 (GSTD3)	AGAP004382-PA	5.1		4.3	glutathione transferase
CUST_3731_PI406199772	CD577517.1		4.7		2.1	cuticle protein
CUST_1088_PI406199772	EE590086.1		4.5		2.6	d7-related 1 protein
CUST_15331_PI426302897	Afun015331	AGAP001039-PB	3.9		29.4	cytochrome p450 307a1
CUST_3736_PI406199772	CD577515.1		3.8		3.3	cuticle protein
CUST_12666_PI426302897	Afun012666	AGAP002429-PA	3.7		3.8	cytochrome p450
CUST_7722_PI426302897	Afun007722	AGAP009850-PA	3.6		2.8	abc transporter
CUST_5005_PI406199798	AGAP003680-RA	AGAP003680-RA	3.5		3.4	abc transporter
CUST_574_PI406199772	EE589639.1		3.2		5.4	d7-related 1 protein
CUST_4392_PI426302897	Afun004392	AGAP008213-PA	2.7		2.6	cytochrome p450
CUST_143_PI426302897	Afun000143	AGAP000818-PA	2.4		2.7	cytochrome p450 cyp9k1
CUST_13218_PI426302897	Afun013218	AGAP000284-PA	2.3		2.3	cytochrome p450
CUST_10836_PI426302897	Afun010836	AGAP006228-PA	2.3		3.7	esterase b1
CUST_45_PI426302897	Afun000045 (GSTe2)	AGAP009194-PA	2.1		12.2	glutathione-s-transferase gst
CUST_13870_PI426302897	Afun013870	AGAP012697-PA	14.8	7.5		sulfotransferase
CUST_12777_PI426302897	Afun012777	AGAP009246-PA	10.3	2.1		cytochrome p450
CUST_9_PI426302915	CYP6M4.seq		9.2	5.3		cytochrome p450
CUST_9492_PI426302897	Afun009492	AGAP001722-PA	8.8	5.9		carboxylesterase
CUST_15523_PI426302897	Afun015523	AGAP010581-PA	5.7	3.1		abc transporter

CUST_979_PI426302897	Afun000979	AGAP006711-PA	4.5	3.7		chymotrypsin 1
CUST_1149_PI406199772	EE590022.1		4.4	2.4		d7-related 1 protein
CUST_3836_PI406199772	CD577459.1		4.3	3.6		cuticle protein
CUST_13272_PI406199769	combined_c6791		4.2	4.8		cytochrome p450
CUST_3830_PI406199772	CD577462.1		4.2	2.7		cuticle protein
CUST_11963_PI426302897	Afun011963	AGAP006220-PA	4.0	2.8		aldehyde oxidase
CUST_4920_PI406199772	BU038983.1		3.8	2.6		cuticle protein
CUST_1150_PI406199772	EE590022.1		3.7	2.3		d7-related 1 protein
CUST_13273_PI406199769	combined_c6791		3.6	4.2		cytochrome p450
CUST_7_PI426302915	CYP6M3.seq		2.9	10.1		cytochrome p450
CUST_1687_PI406199772	EE589439.1		2.9	5.7		d7-related 1 protein
CUST_7127_PI426302897	Afun007127	AGAP009241-PA	2.6	2.4		cytochrome p450
CUST_7008_PI426302897	Afun007008	AGAP012156-PA	2.6	2.2		ABC transporter
CUST_5935_PI406199769	combined_c3002		2.4	2.7		superoxide dismutase
CUST_7482_PI426302897	Afun007482	AGAP002693-PA	2.4	2.3		ABC transporter
CUST_12478_PI426302897	Afun012478	AGAP005757-PA	2.2	3.1		carboxylesterase
CUST_7696_PI406199798	AGAP008141-RA___3R	AGAP008141-RA	2.2	8.5		argininosuccinate lyase
CUST_3394_PI426302897	Afun003394	AGAP000284-PA	2.1	2.4		cytochrome p450
CUST_7499_PI426302897	Afun007499 (GSTD1-5)	AGAP004164-PA	2.1	2.9		glutathione transferase
CUST_27_PI406199775	CYP6P9a			39.4	4.3	cytochrome p450
CUST_30_PI406199775	CYP6P9b			24.0	4.1	cytochrome p450
CUST_25_PI406199775	CYP6P9a			16.3	3.4	cytochrome p450
CUST_1096_PI406199769	combined_c557			11.8	3.8	trypsin
CUST_26_PI406199775	CYP6P9a			9.8	2.7	cytochrome p450
CUST_13614_PI426302897	Afun013614	AGAP003446-PA		8.3	4.0	zinc finger protein 616
CUST_13328_PI426302897	Afun013328	AGAP000384-PA		8.0	2.2	zinc finger protein 650
CUST_8241_PI406199769	combined_c4173			4.8	3.7	glycoprotein 93

CUST_200_PI426302897	Afun000200	AGAP004038-PA	3.5	2.2	chorion peroxidase
CUST_1823_PI406199769	combined_c920		3.4	9.4	glutathione-s-transferase
CUST_431_PI406199788	gb-CYP6M3		2.9	2.6	cytochrome p450
CUST_10614_PI426302897	Afun010614	AGAP006380-PA	2.5	3.0	ABC transporter
CUST_1182_PI406199772	EE589982.1		2.3	2.4	d7-related 1 protein
CUST_7646_PI426302897	Afun007646	AGAP006225-PA	2.0	4.0	aldehyde oxidase

Table S3: Summary statistics for polymorphism of *CYP6P9a* and *CYP6P9b* between the FANG susceptible and the resistant strains from Malawi and Mozambique

Samples	N	S	h	Syn	Nonsyn	π (k)	D (Tajima)	D* (Fu and Li)
CYP6P9A								
Uganda Alive	14	41	7	26	8	0.008 (16.05)	1.07^{ns}	1.4*
Uganda dead	12	15	8	8	6	0.0024 (4.8)	-0.14^{ns}	-0.44^{ns}
All Uganda	26	50	15	30	14	0.0061 (12.1)	-0.35^{ns}	0.88^{ns}
Malawi	20	13	5	4	2	0.001(1.98)	-1.65^{ns}	-1.98 ^{ns}
Total	46	61	20	33	16	0.01(20.1)	1.4 ^{ns}	0.05 ^{ns}
CYP6P9b								
Uganda Alive	16	42	2	22	7	0.003(5.25)	-2.45***	-3.5**
Uganda dead	14	43	3	23	7	0.0029 (6.26)	-2.34***	-3.1**
All Uganda	30	45	4	25	7	0.003 (5.5)	-1.89*	1.19^{ns}
Malawi	20	12	2	6	3	0.0007(1.2)	-2.3**	-3.5**
Total	50	73	6	44	12	0.012(21.43)	0.98^{ns}	1.09^{ns}

N= number of sequences (n); S, number of polymorphic sites; Syn, Synonymous mutations; Nonsyn, Non-synonymous mutations; π, nucleotide diversity (k= mean number of nucleotide differences); Tajima's D and Fu and Li's D statistics, ns, not significant.
